# Editorial: Dynamics of cyclic nucleotide signaling in neurons

**DOI:** 10.3389/fncel.2015.00296

**Published:** 2015-08-03

**Authors:** Pierre Vincent, Nicholas C. Spitzer

**Affiliations:** ^1^Biological Adaptation and Ageing, Centre National de la Recherche Scientifique, UMR 8256Paris, France; ^2^Université Pierre et Marie Curie, UMR 8256Paris, France; ^3^Neurobiology Section, Division of Biological Sciences & Kavli Institute for Brain and Mind, University of California San DiegoLa Jolla, CA, USA

**Keywords:** cyclic GMP, cyclic AMP, G-protein coupled receptors, phosphodiesterases, biosensor imaging, sub-cellular compartmentation

The articles in this Frontiers Research Topic describe how neurons integrate the signals carried by cyclic nucleotide. Three articles present original research, two papers provide provocative opinions about aspects of cyclic nucleotides signaling and four reviews provide timely summary and analysis of the state of the field. The three original articles illustrate how biosensors can reveal the details of signaling cascades in “native” neurons, a major advance compared to the use of heterologous expression systems. Nomura et al. (2014) quantify responses at the level of individual neurons, revealing differences between different cortical regions in the sensitivity to dopamine or noradrenalin. As Polito et al. (2013) demonstrate, spatial resolution also allows analysis of the cAMP signaling cascade in specific neuronal types, a condition which is critical in brain regions where several neuronal types are intermingled, as in the striatum. At higher magnification, biosensor imaging reveals how different sub-cellular compartments differentially integrate the same extracellular signal. This is illustrated by Ladarre et al. who show different responsiveness of axonal and dendritic arbors to cannabinoid drugs (Ladarre et al., 2014). Biosensors have generally been used to study increases in cAMP, typically resulting from the activation of receptors coupled to G_s∕olf_, but this topic also reports effects mediated by G_i_. Nomura et al. show that the positive cAMP/PKA response is moderated by the co-activation of G_i_-coupled receptors coexpressed in the same neuron (Nomura et al., 2014). Ladarre et al. report the effect of CB_1_ receptors that are tonically activated by the endogenous production of endocannabinoids (Ladarre et al., 2014). Finally, biosensor imaging opens the door to studies of the dynamics of signaling by analyzing the functional contribution of the enzymes that control the extent of the cAMP signal. Polito et al. show how phosphodiesterases determine the decay kinetics of transient cAMP responses and how modulation of phosphodiesterase allows for cross-regulation between the cGMP and cAMP pathways (Polito et al., 2013).

In an opinion piece, Iyengar proposes that spatially restricted elevation of cAMP levels and their action on downstream effectors may function as a scaling agent for computations by signaling networks (Iyengar, 2015). Goto, Kamioka, and Matsuda address the problem of control of the Rho-family GTPase, Rac, which controls actin dynamics and morphology, migration, and cytokinesis of neurons and regulates higher brain function (Goto et al., 2014). They suggest that progress in understanding this complex signaling requires simultaneous examination of PKA and Rac activities with FRET biosensors and implement this technology. Review articles address important and rapidly developing aspects of the signaling field. Averaimo and Nicol summarize advances in understanding cAMP signaling and its dynamic interaction with cGAMP and calcium to shape neuronal polarization, transmitter specification, axon guidance, and refinement of neuronal connectivity (Averaimo and Nicol, 2014). Gross, Pugh and Burns summarize the general principles of rod phototransduction and describe recent advances in understanding the dynamics of cGMP during single photon responses (Gross et al., 2015). Recognizing the growing body of evidence for the formation of cAMP gradients and microdomains near the sites of cAMP production, Calebiro and Maiellaro review the methods used for monitoring cAMP and protein kinase A (PKA) signaling in living cells and discuss the major hypotheses on the formation of cAMP/PKA microdomains (Calebiro and Maiellaro, 2014). Finally, Gorshkov and Zhang discuss the design of fluorescent biosensors and describe several of them in detail (Gorshkov and Zhang, 2014). They present examples of the use of cyclic nucleotide fluorescent biosensors to study regulation of neuronal function and consider recent advances in the field. Investigators already in the field as well as those newly attracted to it will find these compact presentations a useful source of new information, ideas, and summaries of the state of the art.

## Funding

PV was supported by Labex Bio-Psy. NS was supported by NIH, the Ellison Medical Foundation and the W. M. Keck Foundation.

### Conflict of interest statement

The authors declare that the research was conducted in the absence of any commercial or financial relationships that could be construed as a potential conflict of interest.
